# Supplementary material for: Degree of adaptive response in urban tolerant birds shows influence of habitat-of-origin
Source: PeerJ. 2014 Mar 13;2:e306. doi: 10.7717/peerj.306 (PMC3961155; doi:10.7717/peerj.306)
Supplement: Supplemental Information 1 — •Supplemental Figure S1. Map of study area showing position within Australia and spread of study sites.•Supplemental Figure S2. Original non-metric multidimensional scaling (NMDS) ordination space for all five bird assemblages in urban Melbourne (from Conole and Kirkpatrick 2011).•Supplemental Table 1. Posterior density for landscape-level preferences of urban adapter bird assemblages (species richness) in Frequency Greenspace bins.•Supplemental Table 2. Posterior density for landscape-level preferences of urban adapter bird assemblages (species richness) in IndexCombined bins.•SUPPLEMENTAL R SCRIPT #1. Script for non-metric multidimensional scaling (NMDS) analysis of urban adapter bird data.•SUPPLEMENTAL R SCRIPT #2. Boxplots of assemblage species richness in Combined Index bins.•SUPPLEMENTAL R SCRIPT #3. ‘bayespref’ analysis of Frequency Greenspace data frame•SUPPLEMENTAL R SCRIPT #4. Plotting Frequency Greenspace preference data from ‘bayespref’ analysis.•SUPPLEMENTAL R SCRIPT #5. Script for a hierarchical agglomerative cluster analysis of ‘habitat-of-origin’ for urban tolerant birds. [file peerj-02-306-s001.pdf]

SUPPLEMENTARY TEXT FOR:

Degree of adaptive response in urban tolerant birds shows influence of habitat-of-origin

Lawrence E. Conole [1,2]

1. School of Geography and Environmental Studies, University of Tasmania, Private Bag 72, Sandy Bay, Tasmania 7002, Australia.

2. Current address: 7 Maxwell Street, Tylden, Victoria 3444, Australia. lconole@gmail.com

The data sets that were utilised in (Conole, 2013) were derived and refined as described below. Most of these procedures were initially carried out to support analyses reported in Conole and Kirkpatrick (2011).

Study area

The study area is metropolitan Melbourne; capital city of the State of Victoria in coastal southeastern Australia, within a 50 km radius of its Central Business District (37°49'S 144°58'E). The study area excludes the sea, but includes areas not yet urbanised. The total area of metropolitan Melbourne is approximately 880,000 ha, with a population in 2007 of approximately 3.8 million people (DPCD 2008). Suburbs, with detached single dwellings in gardens dominated by plant species exotic to Melbourne, cover most of the above area. Semi-natural remnants of native vegetation are scattered within the bounds of the urban area, which also contains many parks and gardens planted with exotic plant species. Trees are planted in most streets; these tend to be native to Australia, but not to the Melbourne region (Frank, Waters, Beer, & May, 2006). The original vegetation of Melbourne and the native vegetation that survives on its margins is highly varied, this variation being related to soils, which range from highly fertile black, cracking clays to highly infertile deep leached sands, and annual rainfall, which ranges from 540 to 1,000 mm from the west to the east.

Atlas data

Approximately 220,000 records of 292 species of birds from 11,434 surveys were extracted from the Birds Australia 'New Atlas of Australian Birds' project database (hereafter 'the Atlas') (Barrett, Silcocks, Barry, Cunningham, & Poulter, 2003). The Atlas database contains four types of record: 2-ha search for 20 minutes; small area search (within 500 m of a central point); large area search (within 5 km of a central point); and, incidental observations of individual species from a single point (Barrett et al., 2003).

Each survey represents a list of species for a defined area and time (ranging from 20 minutes to one month), with geographic co-ordinates. All data were collected between 1998 and 2002. Data initially extracted for this study included 4,221 2-ha searches, 4,993 small area searches, 793 large area searches, and 1,427 incidental observations, and were compiled in a matrix as species and their relative abundance (number of surveys in which a species was recorded in a cell divided by the total number of surveys conducted in the cell) by site.

Using ArcMap GIS, a 1 x 1 km grid based on that developed by the Australian Research Centre for Urban Ecology (ARCUE) (Hahs & McDonnell, 2006) was intersected with Atlas records to produce a matrix of grid cells by species presence/absence. All surveys were assigned to the grid cell in which the central geographic coordinates fell, regardless of survey spatial or temporal scale. It was assumed that most large area searches (6.9% of the surveys in the unfiltered data set) referred to areas of between 500 – 2,000 m diameter, and therefore could reasonably be assigned to 1 x 1 km grid cells within which the central coordinates fell.

Estimated sampling completeness

As there is a likelihood that less abundant species may be missed where sampling effort is lower, leading to uneven representation of species (Watson, 2003), a measure of estimated sampling completeness was calculated for each of the grid cells. This enabled an assessment of the evenness of sampling, and for unrepresentative samples to be removed from the data to be analysed.

First, the predicted number of species (S_{Chao2}) was calculated for each cell in a 66 km x 65 km grid, using the $Chao_2$ formula (Chao, 1987) (Supplementary Formula 1), where S_{obs} equals the number of species observed, Q_1 the number of unique records (species observed only once at a site during surveys), and Q_2 the number of doubletons (species observed only twice). S_{Chao2} is the estimated total number of species present at survey sites, including those not found during surveys.

$$S_{Chao2} = S_{Obs} + \frac{Q_1^2}{2Q_2}$$

Supplementary Formula 1: Chao2

From these calculations a standardized measure of sampling completeness (%Completeness) was also calculated for each grid cell, with observed species richness (S_{obs}) as a proportion of predicted species richness (S_{Chao2}) (Peterson & Slade, 1998).

Data organisation

Several assumptions were made about species to be excluded from analyses, and species were not included in grid cell totals and were eliminated from further analyses if any of the following exclusion criteria were met: (i) constituted fewer than five records in the total dataset; (ii) was an irregular or vagrant species to the area or feral species not yet naturalised, determined from the literature (Barrett et al., 2003); or, (iii) were seabird, waterbird, and nocturnal species, except the Tawny Frogmouth *Podargus strigoides*, from the orders or families: Anseriformes, Podicipediformes, Strigiformes, Eurostopodidae, Aeogothelidae, Procellariformes, Spenisciformes, Phalacrocaraciformes, Ciconiiformes, Gruiformes, Charadriiformes (sensu Christidis & Boles, 2008). A final list of 141 species (hereafter 'the filtered species list') was retained for further analysis.

Grid cells were eliminated from further analyses if any of the following exclusion criteria were met: (i) ≤ 1 surveys in the cell; (ii) %Completeness < 50%; (iii) land area < 25% of the cell; or (iv) a high proportion of singleton records (>50%) and/or no doubleton records (indicating skewed data collection, e.g. single-species or other narrowly targeted surveys). A final list of 390 grid cells was retained for further analysis.

Bird species were classified into foraging guilds using a modified scheme for southern Victorian species (Mac Nally, 1994). Mac Nally's (1994) 'Hawker' and 'Sweeper' categories were combined to make 'Hawker/Sweeper', 'Wood Searcher' and 'Bark Prober' combined to make 'Wood Searcher/Bark Prober', and the categories of 'Raptor' and 'Frugivore' were added. Species not classified by Mac Nally (1994) were classified according to data contained in the 'Handbook of Australian, New Zealand and Antarctic Birds' (HANZAB) (Higgins, 1999; Higgins & Davies, 1996; Higgins & Peter, 2002; Higgins, Peter, & Cowling, 2006; Higgins, Peter, & Steele, 2001; Marchant & Higgins, 1993). Nest substrate and dispersal groupings were also assigned from data contained in HANZAB. Data on bird size was tabulated as maximum mass (in grams) from HANZAB. Systematics and nomenclature of birds follow Christidis and Boles (2008).

Spatial data on the degree of urbanisation of the study area employed in this study were developed at ARCUE and are discussed in detail by Hahs and McDonnell (Hahs & McDonnell, 2006); a brief summary follows. People per square kilometre (People/km^2) is the total number of people in census collection districts (Australian Bureau of Statistics 2003). Dwellings per square kilometre (Dwellings/ km^2) is the total number of dwellings in census collection districts (Australian Bureau of Statistics 2003). Frequency Greenspace is the reciprocal of the average amount of impervious surface calculated at the sub-pixel level from the impervious surface fraction image created during the spectral mixture analysis of the 2000 Landsat ETM+ image (Hahs & McDonnell, 2006). Combined index ($Index_{Combined}$) is the

average value of $Index_{Image}$ and $Index_{Census}$; where $Index_{Image}$ is calculated from fraction images produced by the spectral mixture analysis of the 2000 Landsat ETM+ image, and $Index_{Census}$ = the total number of people multiplied by the proportion of males employed in non-agricultural work, as enumerated in the 2001 census (Hahs & McDonnell, 2006). Combined index was found to be a useful measure for determining the level of urbanisation represented by a combination of demographic and spatial data (Hahs & McDonnell, 2006).

Metrics were calculated for all cells in the 65 x 66 km ARCUE grid (Hahs & McDonnell, 2006) (Amy Hahs pers. comm, 25 May 2007).

Data analysis

All statistical analyses were performed in R (R Core Team, 2013), using core functions and procedures from the community ecology package 'vegan' (Oksanen et al., 2013). Figures were drawn using R core functions, and the 'PBSmapping' (Schnute et al., 2013) and 'sp' (Bivand, Pebesma, & Gómez-Rubio, 2013) packages.

The data for cluster analysis consisted of a standard row by column 'r x c' array, with sites as rows, species as columns, and relative abundance (% incidence in surveys conducted in each cell) data for species occurring in each grid cell. A Bray-Curtis distance matrix was prepared, and groups of species were formed by hierarchical agglomerative clustering using Ward's algorithm performed on the distance matrix, as a function of their similarity in distribution and relative abundance. Following González Oreja et al., (González-Oreja, Bonache-Regidor, Buzo-Franco, la Fuente Díaz Ordaz, & Hernández Satín, 2007), an assemblage is a cluster of species separated from all other such clusters by an ecological distance greater than the greatest distance between the two most disparate members of the clade. Where significant sub-structuring in the dendrogram coincided with diagnosable trends in the environmental and demographic data, sub-assemblages were recognized. Assemblages were named using Blair's (1996) standard nomenclature, in keeping with its wide use in the urban bird ecology literature (Chace & Walsh, 2006).

The species and grid cells were ordinated by global non-metric multidimensional scaling (NMDS) methods, using the 'vegan' package (Oksanen et al., 2013). A two-dimensional solution using the Wisconsin square-root transformation and Bray-Curtis coefficients as a measure of dissimilarity in species composition between the sample plots was chosen. Vectors for seven variables were fitted to both the species and grid cell two-dimensional ordination space using the procedure, 'envfit', in 'vegan', and the species ordination space was plotted in an ordination graphic.

Each grid cell was attributed to the bird cluster that had the highest proportion of its total number of species within it, except for 13 cells out of 390 (3.3%), which had equal numbers of cluster 2a and 2b species present.

A Kruskal-Wallis test was used to test whether these five groups of grid cells differed in a simple measure of urbanisation intensity, People/km^2. A Kruskal-Wallis test was also used to test whether these five groups of grid cells differed in longitude (indicating their position on a west to east environmental gradient in Melbourne), and to test whether bird mass differed significantly between the five groups. The Mann-Whitney U-test was used to determine which means were significantly different from others. As we were principally interested in the 'comparisonwise error rate' rather than the 'experimentise error rate', an α correction (such as Bonferroni) for multiple comparison testing was judged to be unnecessary (Bender & Lange, 2001).

The assemblage members were then allocated to the categories of urban exploiter, suburban adapter, or urban avoider (Blair, 1996) on the basis of their membership of the clusters associated with different levels of urbanisation intensity. This process differs from the method employed in some other studies, in which the urban bird classes were aligned a priori with predetermined classes of urbanisation intensity (Croci, Butet, & Clergeau, 2008; White, Antos, Fitzsimons, & Palmer, 2005).

References

Barrett, G. W., Silcocks, A., Barry, S., Cunningham, R. B., & Poulter, R. (2003). *The New Atlas of Australian Birds*. Hawthorn East: Royal Australasian Ornithologists Union.

Bender, R., & Lange, S. (2001). Adjusting for multiple testing - when and how? *Journal of Clinical Epidemiology*, *54*, 343–349. Retrieved from http://ajph.aphapublications.org/doi/pdf/10.2105/AJPH.86.5.726

Bivand, R. S., Pebesma, E., & Gómez-Rubio, V. (2013). *Applied Spatial Data Analysis with R*. New York, NY: Springer New York. doi:10.1007/978-1-4614-7618-4

Blair, R. B. (1996). Land use and avian species diversity along an urban gradient. *Ecological Applications*, *6*, 506–519.

Chace, J. F., & Walsh, J. J. (2006). Urban effects on native avifauna: a review. *Landscape and Urban Planning*, *74*(1), 46–69. doi:10.1016/j.landurbplan.2004.08.007

Chao, A. (1987). Estimating the population size for capture-recapture data with unequal catchability. *Biometrics*, *43*, 783–791.

Christidis, L., & Boles, W. E. (2008). *Systematics and taxonomy of Australian birds*. Collingwood: CSIRO.

Conole, L. E. (2013). Habitat-of-origin predicts degree of adaptation in urban tolerant birds. *PeerJ PrePrints*. doi:10.7287/peerj.preprints.156v2

Conole, L. E., & Kirkpatrick, J. B. (2011). Functional and spatial differentiation of urban bird assemblages at the landscape scale. *Landscape and Urban Planning*, *100*(1-2), 11–23. doi:10.1016/j.landurbplan.2010.11.007

Croci, S., Butet, A., & Clergeau, P. (2008). Does Urbanization Filter Birds on the Basis of Their Biological Traits? *The Condor*, *110*(2), 223–240. doi:10.1525/cond.2008.8409

DPCD (2008). Victoria in Future 2008 – detailed files. Melbourne: Victorian Government Department of Planning and Community Development. http://www.dse.vic.gov.au/DSE/dsenres.nsf/childdocs/-4CA023FAC31D9D5CCA256D650016CB01-C70997566F01CABDCA256D6500039059-775206E3E0281595CA256F0E0013C1FB?open [Accessed 16 May 2009]

Frank, S., Waters, G., Beer, R., & May, P. G. (2006). An analysis of the street tree population of Greater Melbourne at the beginning of the 21st Century. *Arboriculture and Urban Forestry*, *32*(4), 155.

González-Oreja, J. A., Bonache-Regidor, C., Buzo-Franco, D., la Fuente Díaz Ordaz, de, A. A., & Hernández Satín, L. (2007). Caracterización Ecológica De La Avifauna De Los Parques Urbanos De La Ciudad De Puebla (México). *Ardeola*, *54*(1), 53–67.

Hahs, A. K., & McDonnell, M. J. (2006). Selecting independent measures to quantify Melbourne's urban–rural gradient. *Landscape and Urban Planning*, *78*(4), 435–448. doi:10.1016/j.landurbplan.2005.12.005

Higgins, P. J. (Ed.). (1999). *Handbook of Australian, New Zealand and Antarctic Birds. Volume 4: Parrots to Dollarbird.* (Vol. 4). Melbourne: Oxford University Press.

Higgins, P. J., & Davies, S. J. J. F. (Eds.). (1996). *Handbook of Australian, New Zealand and Antarctic Birds. Volume 3: Snipe to Pigeons* (Vol. 3). Melbourne: Oxford University Press.

Higgins, P. J., & Peter, J. M. (Eds.). (2002). *Handbook of Australian, New Zealand and Antarctic Birds. Volume 6: Pardalotes to Shrike-thrushes.* (Vol. 6). Melbourne: Oxford University Press.

Higgins, P. J., Peter, J. M., & Cowling, S. J. (Eds.). (2006). *Handbook of Australian, New Zealand and Antarctic Birds. Volume 7: Boatbill to Starlings.* (Vol. 7). Melbourne: Oxford University Press.

Higgins, P. J., Peter, J. M., & Steele, W. K. (Eds.). (2001). *Handbook of Australian, New Zealand and Antarctic Birds. Volume 5: Tyrant-flycatchers to Chats.* (Vol. 5). Melbourne: Oxford University Press.

Mac Nally, R. (1994). Habitat-specific guild structure of forest birds in south-eastern Australia: a regional scale perspective. *Journal of Animal Ecology*, 988–1001.

Marchant, S., & Higgins, P. J. (Eds.). (1993). *Handbook of Australian, New Zealand and Antarctic Birds. Volume 2: Raptors to Lapwings* (Vol. 2). Melbourne: Oxford University Press.

Oksanen, J., Guillaume Blanchet, F., Kindt, R., Legendre, P., Minchin, P. R., O'Hara, R. B., et al. (2013). vegan: Community Ecology Package. Comprehensive R Archive Network.

Peterson, A., & Slade, N. (1998). Extrapolating inventory results into biodiversity estimates and the importance of stopping rules. *Diversity and Distributions*, *4*(3), 95–105. doi:10.1046/j.1365-2699.1998.00021.x

R Core Team. (2013, October 3). R: A language and environment for statistical computing. Vienna: R Foundation for Statistical Computing.

Schnute, J. T., Boers, N., Haigh, R., Grandin, C., Johnson, A., Wessell, P., & Antonio, F. (2013). PBSmapping: Mapping Fisheries Data and Spatial Analysis Tools. Retrieved from http://CRAN.R-project.org/package=PBSmapping

Watson, D. M. (2003). The “standardized search”: An improved way to conduct bird surveys. *Austral Ecology*, *28*(5), 515–525. Retrieved from http://onlinelibrary.wiley.com/doi/10.1046/j.1442-9993.2003.01308.x/full

White, J. G., Antos, M. J., Fitzsimons, J. A., & Palmer, G. C. (2005). Non-uniform bird assemblages in urban environments: the influence of streetscape vegetation. *Landscape and Urban Planning*, *71*(2-4), 123–135. doi:10.1016/j.landurbplan.2004.02.006

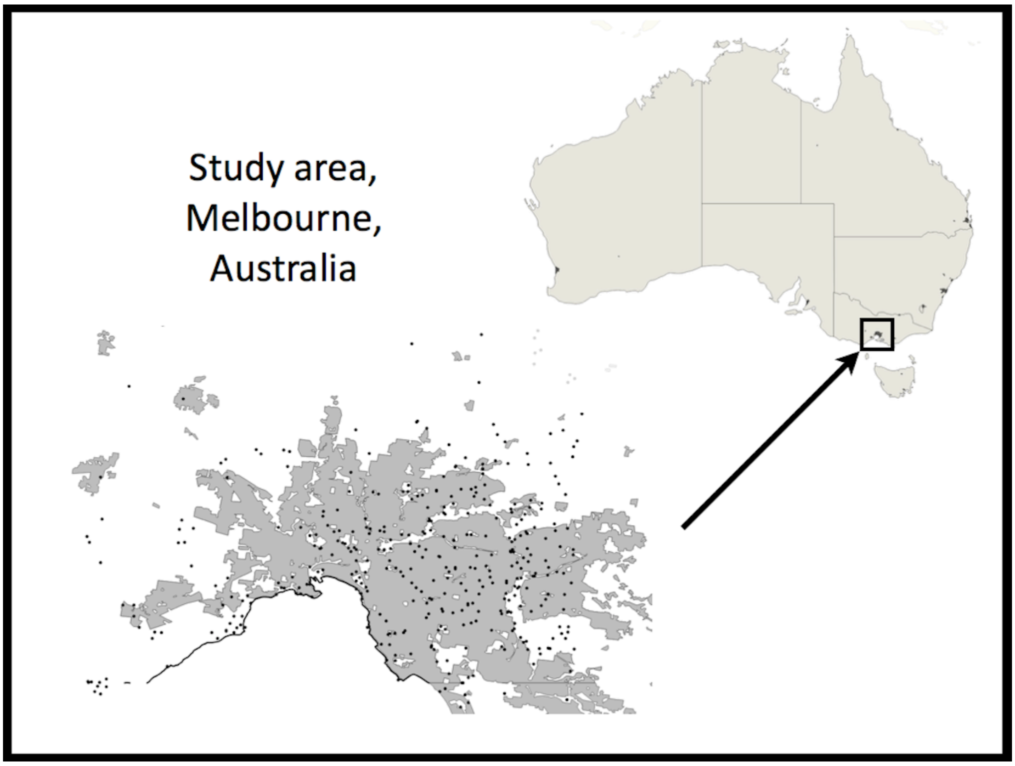

Supplementary Figure S1: Map of study area showing position within Australia and spread of study sites.

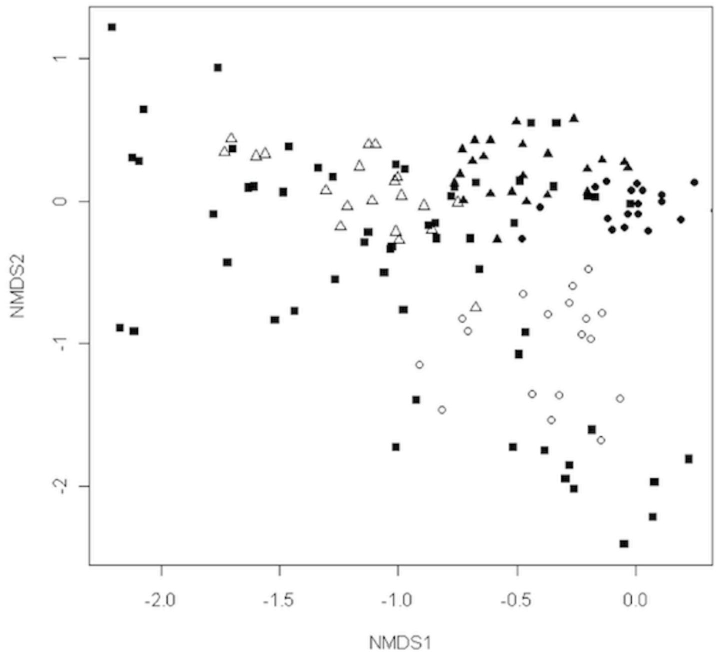

Supplementary Figure S2: Original non-metric multidimensional scaling (NMDS) ordination space for all five bird assemblages in urban Melbourne (from Conole and Kirkpatrick 2011).

Supplementary Table 1: Posterior density for landscape-level preferences of urban adapter bird assemblages (species richness) in Frequency Greenspace bins

Frequency Greenspace		Urban adapter		Urban exploiter	
**Bin name**	**Range**	**Median preference**	**Credible intervals**	**Median preference**	**Credible intervals**
2	20 – 29.9	0.206	0.126, 0.292	0.794	0.708, 0.874
3	30 – 39.9	0.279	0.222, 0.347	0.721	0.653, 0.778
4	40 – 49.9	0.289	0.268, 0.33	0.701	0.67, 0.732
5	50 – 59.9	0.396	0.367, 0.423	0.604	0.577, 0.633
6	60 – 69.9	0.46	0.424, 0.494	0.54	0.506, 0.576

Supplementary Table 2: Posterior density for landscape-level preferences of urban adapter bird assemblages (species richness) in $\text{Index}_{\text{Combined}}$ bins

$\text{Index}_{\text{Combined}}$		Urban adapter		Urban exploiter	
**Bin name**	**Range**	**Median preference**	**Credible intervals**	**Median preference**	**Credible intervals**
0	0 – 4.9	0.252	0.144, 0.392	0.748	0.608, 0.856
1	5.0 – 9.9	0.346	0.263, 0.435	0.654	0.565, 0.737
2	10 – 14.9	0.418	0.359, 0.485	0.582	0.515, 0.641
3	15 – 19.9	0.4	0.34, 0.469	0.6	0.531, 0.66
4	20 – 24.9	0.466	0.407, 0.524	0.534	0.476, 0.593
5	25 – 29.9	0.462	0.397, 0.52	0.538	0.48, 0.603
6	30 – 34.9	0.436	0.398, 0.476	0.569	0.523, 0.602
7	35 – 39.9	0.431	0.398, 0.466	0.569	0.534, 0.602
8	40 – 44.9	0.345	0.311, 0.378	0.655	0.622, 0.689
9	45 – 50	0.243	0.154, 0.34	0.757	0.66, 0.846

SUPPLEMENTARY R SCRIPT #1

Script for non-metric multidimensional scaling (NMDS) analysis of urban adapter bird data

This is an R Mardkown file which contains a 'vegan' script for a non-metric multidimensional scaling ordination for the urban adapter bird assemblage that I ran in:

Conole, L. E. (2013). Habitat-of-origin predicts degree of adaptation in urban tolerant birds. *PeerJ PrePrints*. doi:10.7287/peerj.preprints.156v2. https://peerj.com/preprints/156v2

```
library(vegan)
```

```
## Loading required package: permute
## Loading required package: lattice
## This is vegan 2.0-10
```

```
clade2a <- read.table("clade2a.RA.txt", header = T)
head(clade2a)
```

```
##      Australian.Raven Bell.Miner Blackfaced.CuckooShrike Brown.Thornbill
## AA45           0.0000          0                  0.0000               0
## AC44           0.0290          0                  0.0010               0
## AD36           0.0000          0                  0.0238               0
## AD46           0.0435          0                  0.0000               0
## AE45           0.0000          0                  0.0000               0
## AF34           0.0000          0                  0.0000               0
##      Common.Bronzewing Crimson.Rosella Eastern.Rosella Eastern.Spinebill
## AA45                 0               0               0                 0
## AC44                 0               0               0                 0
## AD36                 0               0               0                 0
## AD46                 0               0               0                 0
## AE45                 0               0               0                 0
## AF34                 0               0               0                 0
##      Grey.Butcherbird Grey.Currawong Grey.Fantail Laughing.Kookaburra
## AA45                0              0       0.0000                   0
## AC44                0              0       0.0290                   0
## AD36                0              0       0.0000                   0
## AD46                0              0       0.0000                   0
## AE45                0              0       0.0278                   0
## AF34                0              0       0.0000                   0
##      Noisy.Miner Pied.Currawong Rainbow.Lorikeet Silvereye
## AA45           0          0.000           0.0400     0.000
## AC44           0          0.001           0.0050     0.027
## AD36           0          0.000           0.0476     0.000
## AD46           0          0.000           0.0000     0.000
## AE45           0          0.000           0.0278     0.000
```

```
## AF34           0          0.000           0.0769     0.000
##      Spotted.Pardalote Sulphurcrested.Cockatoo Superb.Fairywren
## AA45             0.000                  0.0000           0.0400
## AC44             0.011                  0.0000           0.0390
## AD36             0.000                  0.0238           0.0238
## AD46             0.000                  0.0000           0.0000
## AE45             0.000                  0.0000           0.0833
## AF34             0.000                  0.0385           0.0000
##      Tawny.Frogmouth Whitebrowed.Scrubwren Yellowtailed.BlackCockatoo
## AA45               0                0.0000                          0
## AC44               0                0.0000                          0
## AD36               0                0.0238                          0
## AD46               0                0.0000                          0
## AE45               0                0.0000                          0
## AF34               0                0.0000                          0
##      Ganggang.Cockatoo
## AA45                 0
## AC44                 0
## AD36                 0
## AD46                 0
## AE45                 0
## AF34                 0
```

In the data frame 'clade2a', columns are species and rows are sites. The values in this 'r x c' frame are relative abundance of each species at each site.

This script runs a three dimensional NMDS ordination.

```
# run NMDS 3D
clade2a.mds <- metaMDS(clade2a, distance = "bray", k = 3, zerodist = "add",
    autotransform = TRUE, noshare = 0.1, wascores = TRUE, expand = TRUE, trace = 1,
    plot = FALSE)
```

```
## Zero dissimilarities changed into  0.02519
## Run 0 stress 0.1585
## Run 1 stress 0.1576
## ... New best solution
## ... procrustes: rmse 0.01035  max resid 0.1198
## Run 2 stress 0.1623
## Run 3 stress 0.1574
## ... New best solution
## ... procrustes: rmse 0.01515  max resid 0.1849
## Run 4 stress 0.16
## Run 5 stress 0.1585
## Run 6 stress 0.1585
## Run 7 stress 0.1588
## Run 8 stress 0.1575
## ... procrustes: rmse 0.01431  max resid 0.1854
## Run 9 stress 0.1648
```

```
## Run 10 stress 0.1616
## Run 11 stress 0.1603
## Run 12 stress 0.1574
## ... New best solution
## ... procrustes: rmse 0.01562  max resid 0.186
## Run 13 stress 0.1589
## Run 14 stress 0.1662
## Run 15 stress 0.1586
## Run 16 stress 0.1599
## Run 17 stress 0.1573
## ... New best solution
## ... procrustes: rmse 0.002593  max resid 0.02866
## Run 18 stress 0.1574
## ... procrustes: rmse 0.006134  max resid 0.06709
## Run 19 stress 0.1606
## Run 20 stress 0.1575
## ... procrustes: rmse 0.01537  max resid 0.1861
```

Plotting the first two axes.

```
# plot 2D NMDS - first 2 axes
ordiplot(clade2a.mds, type = "none", main = "Urban adapter birds - assemblage 2a")
points(clade2a.mds, "sites", pch = 21, col = "black", bg = "black")
text(clade2a.mds, "species", col = "blue", cex = 0.5)
```

A suite of environmental factors were fitted to the ordination. Columns in 'envar.clade2a' represent parameter names and rows represent sites. The values represented by column names are as follows:

- X = longitude in decimal degrees
- Y = latitude in decimal degrees (negative values indicate southern hemisphere)
- IndComb = the combined index, an index of urbanisation intensity
- Peop = density of people (per square kilometre)
- Dwell - density of dwellings (per square kilometre)
- Fr_Green = frequency greenspace (reciprocal of density impervious surfaces)
- LC_Rich = land cover richness
- LC_Dom = land cover dominance
- PC_URB = percent (%) urban
- Sobs = observed bird species richness (all assemblages)
- Sobs.2a - observed species richness of urban adapter birds
- Sobs.2b - observed species richness of urban exploiter birds
- Arbor = an index of 'arborisation', indicating woodiness of former native vegetation prior to urbanisation

More detailed discussion of these parameters can be found in:

Conole, L. E., & Kirkpatrick, J. B. (2011). Functional and spatial differentiation of urban bird assemblages at the landscape scale. *Landscape and Urban Planning*, 100(1-2), 11–23. doi:10.1016/j.landurbplan.2010.11.007

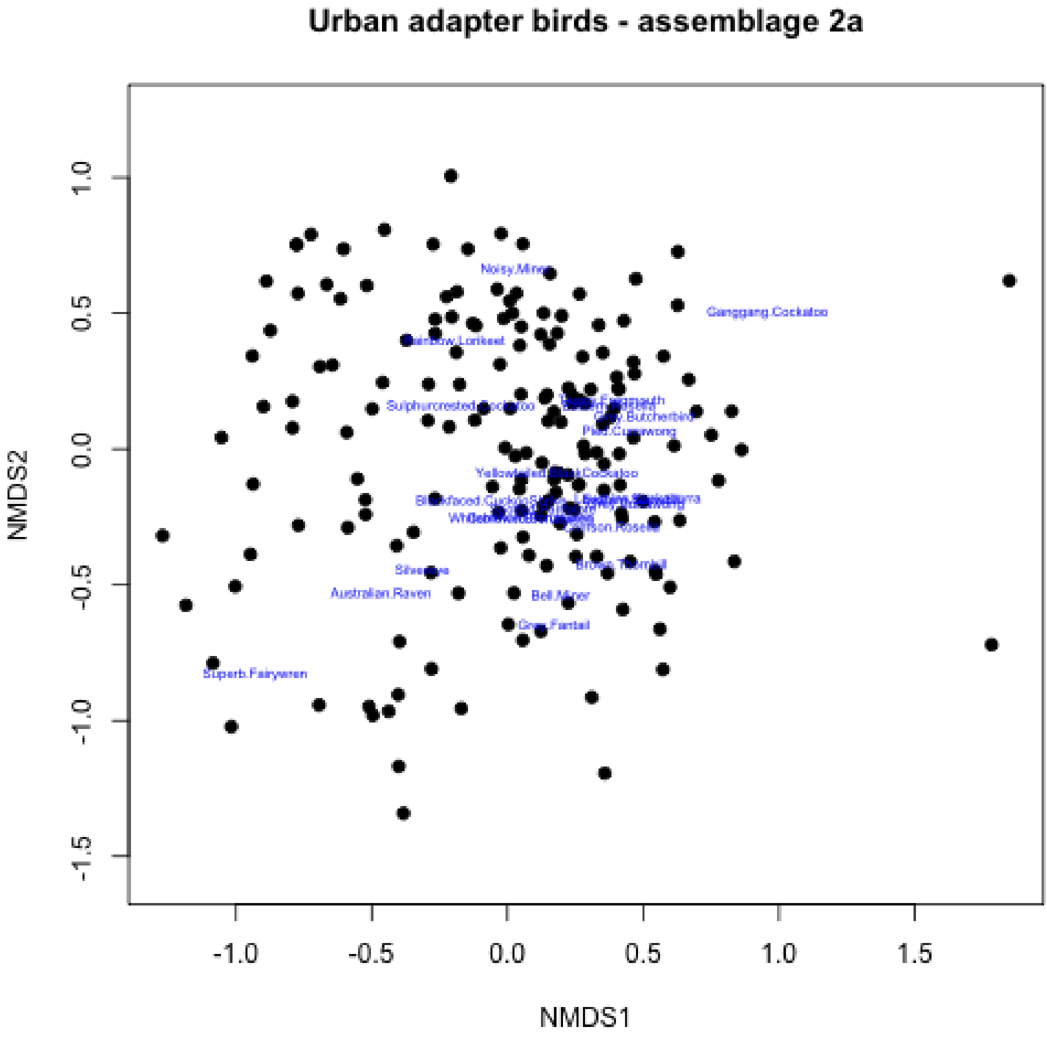

Figure 1: Supplementary R Script Figure 1. Plot first two dimensions of NMDS ordination

```r
# load envar
envar.clade2a <- read.table("qgis.envar.clade2a.txt", header = T)
ef2a <- envfit(clade2a.mds, envar.clade2a, permu = 1000)
```

Re-plotting the NMDS ordination space with fitted vectors shown as arrows. Directional difference shows different gradients. Arrow length indicates strength of relationship to the data.

```r
plot(clade2a.mds, display = "sites", main = "Urban adapter birds - assemblage 2a")
plot(ef2a, p.max = 0.05, col = "black")
```

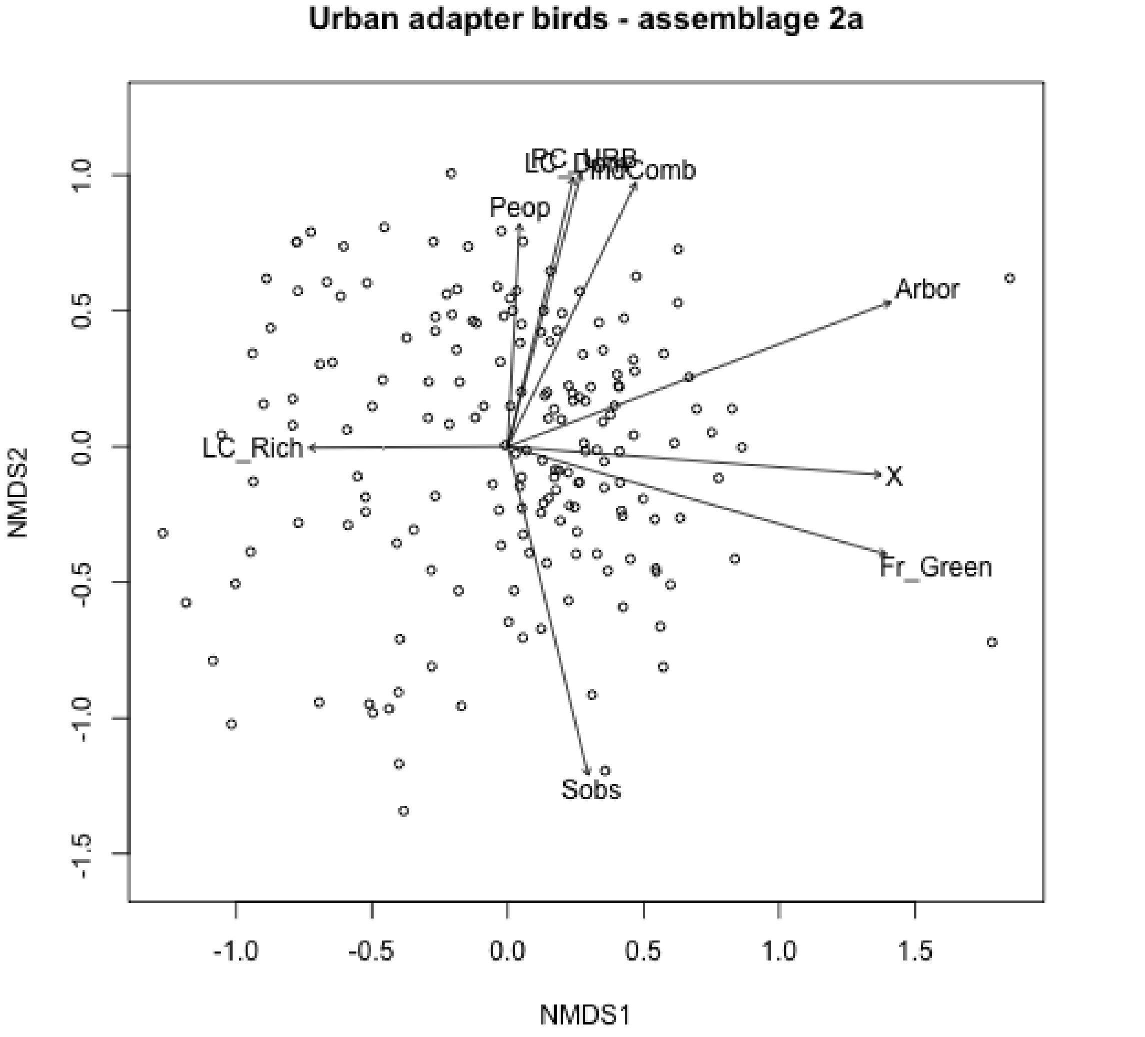

Figure 2: Supplementary R Script Figure 2. Plot NMDS ordination with vectors

```
ef2a
```

```
##
## ***VECTORS
##
##             NMDS1  NMDS2   r2 Pr(>r)
## X          0.997 -0.076 0.12  0.001 ***
## Y          0.608 -0.794 0.01  0.528
## IndComb    0.437  0.899 0.07  0.001 ***
## Peop       0.053  0.999 0.04  0.021 *
## Dwell      0.002  1.000 0.03  0.086 .
## Fr_Green   0.962 -0.274 0.13  0.001 ***
## LC_Rich   -1.000 -0.006 0.03  0.043 *
## LC_Dom     0.237  0.971 0.06  0.003 **
## PC_URB     0.259  0.966 0.07  0.003 **
## Sobs       0.238 -0.971 0.10  0.001 ***
## Arbor      0.936  0.353 0.14  0.001 ***
## ---
## Signif. codes:  0 '***' 0.001 '**' 0.01 '*' 0.05 '.' 0.1 ' ' 1
## P values based on 1000 permutations.
```

The matrix of vectors, and their significance in explaining the ordination, is shown above.

SUPPLEMENTARY R SCRIPT #2

Boxplots of assemblage species richness in Combined Index bins

This is an R Markdown documentwhich contains a script for plotting boxplots of urban tolerant bird assemblage species richness binned by a measure of urbanisation intensity (Combined Index) that I ran in:

Conole, L. E. (2013). Habitat-of-origin predicts degree of adaptation in urban tolerant birds. *PeerJ PrePrints*. doi:10.7287/peerj.preprints.156v2. link

Read in data from tab-delimited text file and eyeball the first few lines of the resultant dataframe.

```
ic <- read.delim("ic.2ab.txt", header = T)
head(ic)
```

```
##   X adapter exploiter
## 1 1       2         7
## 2 1      11        19
## 3 1       2        13
## 4 1       6         9
## 5 1       5        16
## 6 1       0        11
```

This script plots two series of boxplots (one for adapters, one for exploiters), organised in 10 bins of urbanisation intensity (Combined Index), side-by-side in a single plotting space:

```
par(mfrow = c(1, 2))
boxplot(ic$adapter ~ ic$X, boxwex = 0.45, ylab = "Assemblage species richness",
    xlab = "Adapters")
boxplot(ic$exploiter ~ ic$X, boxwex = 0.45, ylab = "", xlab = "Exploiters")
```

An identical workflow was created for plotting the equivalent Frequency Greenspace data.

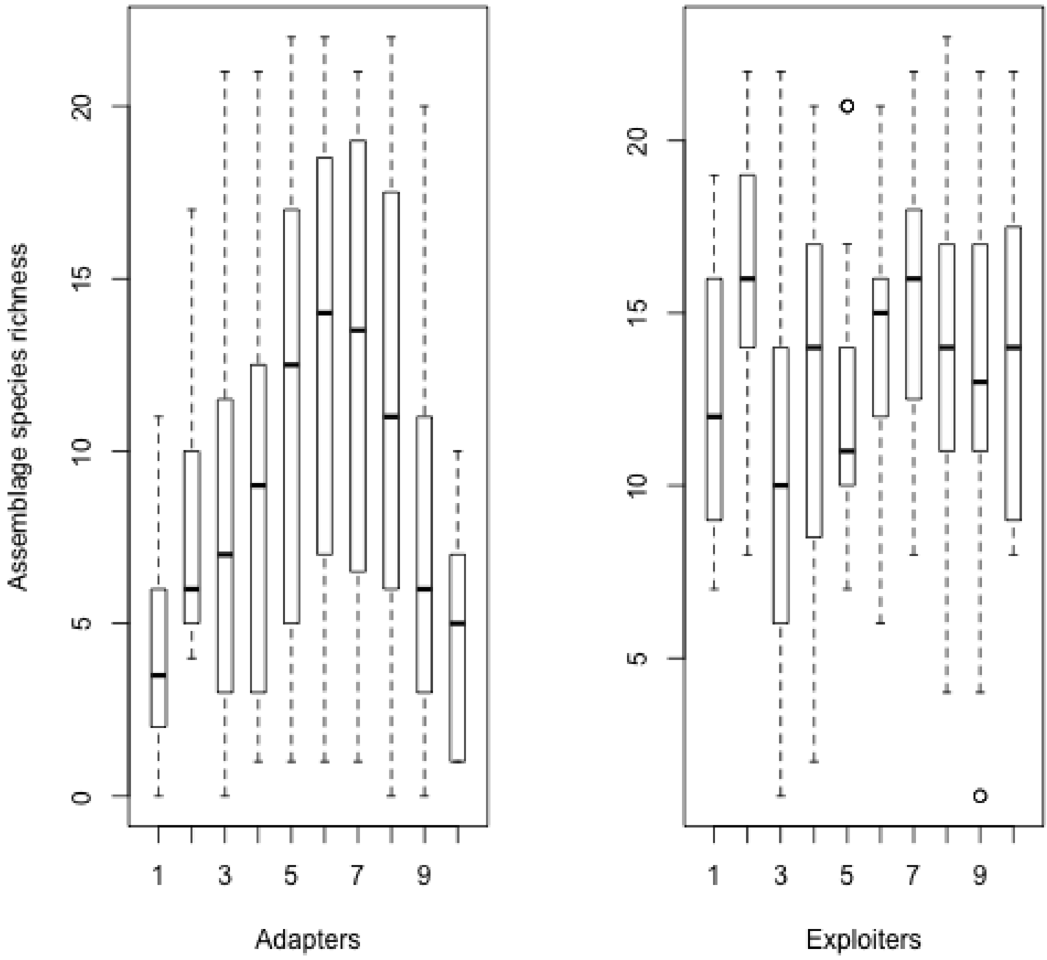

Figure 1: Supplementary R Script Figure 3. Side-by-side box plots of urban tolerant bird assemblage species richness

SUPPLEMENTARY R SCRIPT #3:

'bayespref' analysis of Frequency Greenspace data frame

This is an R Mardkown file which sets out the basic 'bayespref' analysis that I ran on Grequency Greenspace data and urban tolerant birds in:

Conole, L. E. (2013). Habitat-of-origin predicts degree of adaptation in urban tolerant birds. PeerJ PrePrints. doi:10.7287/peerj.preprints.156v2. https://peerj.com/preprints/156v2

```
library(bayespref)
```

```
## Loading required package: coda
## Loading required package: lattice
## Loading required package: MASS
## Loading required package: MCMCpack
## ##
## ## Markov Chain Monte Carlo Package (MCMCpack)
## ## Copyright (C) 2003-2014 Andrew D. Martin, Kevin M. Quinn, and Jong Hee Park
## ##
## ## Support provided by the U.S. National Science Foundation
## ## (Grants SES-0350646 and SES-0350613)
## ##
## Loading required package: RColorBrewer
```

Read in data and eyeball the first few lines of the resultant data frame.

```
fg <- read.table("fg.2ab.txt", header = T)
head(fg)
```

```
##   pop adapter exploiter
## 1   1       0         0
## 2   1       0         0
## 3   1       0         0
## 4   1       0         0
## 5   1       0         0
## 6   2       0         0
```

In the data frame 'fg', column headings and the data to which they refer are as follows:

- pop indicates population; in this case referring to 7 binned intervals of Frequency Greenspace in the larger data set
- adapter indicates species richness of urban adapter birds at a given site within the Frequency Greenspace bin

- exploiter indicates species richness of urban exploiter birds at a given site within the Frequency Greenspace bin

In this example I have set the number of MCMC steps at 10,000 and burn-in generations to 1,000, as for the analysis shown in the manuscript.

```
fgm <- as.matrix(fg)
fgpref <- bayesPref(pData = fgm, mcmcL = 10000, pops = T, dicburn = 1000, dirvar = 20)
```

```
## current mcmc step: 1;   p(M|D):  -1333
## current mcmc step:  100 ;  p(M|D):  -413
## current mcmc step:  200 ;  p(M|D):  -423.2
## current mcmc step:  300 ;  p(M|D):  -456.2
## current mcmc step:  400 ;  p(M|D):  -427.7
## current mcmc step:  500 ;  p(M|D):  -452.6
## current mcmc step:  600 ;  p(M|D):  -451.6
## current mcmc step:  700 ;  p(M|D):  -423.2
## current mcmc step:  800 ;  p(M|D):  -417.4
## current mcmc step:  900 ;  p(M|D):  -422.4
## current mcmc step:  1000 ;  p(M|D):  -415.3
## current mcmc step:  1100 ;  p(M|D):  -413.5
## current mcmc step:  1200 ;  p(M|D):  -433.1
## current mcmc step:  1300 ;  p(M|D):  -460
## current mcmc step:  1400 ;  p(M|D):  -425.8
## current mcmc step:  1500 ;  p(M|D):  -456.9
## current mcmc step:  1600 ;  p(M|D):  -448.9
## current mcmc step:  1700 ;  p(M|D):  -476.8
## current mcmc step:  1800 ;  p(M|D):  -431.6
## current mcmc step:  1900 ;  p(M|D):  -453.5
## current mcmc step:  2000 ;  p(M|D):  -445.6
## current mcmc step:  2100 ;  p(M|D):  -444.4
## current mcmc step:  2200 ;  p(M|D):  -431.8
## current mcmc step:  2300 ;  p(M|D):  -418.5
## current mcmc step:  2400 ;  p(M|D):  -416
## current mcmc step:  2500 ;  p(M|D):  -414.8
```

Output truncated at step 2,500 (of 10,000)

Setting the proposal distance in the MCMC (dirvar) to the default of 2 resulted in poor mixing of MCMC chains, and so after a number of trials a satisfactory mixing of chains was achieved by setting dirvar=20.

The plot shown below is a representation of chain mixing from plotting MCMC steps against population preference.

```
plot(fgpref[[2]]$PopPref[1, ], xlab = "MCMC step", ylab = "PopPref")
```

Now satisfied with the mixing of the MCMC, examine the parameter estimates provided by the analysis. The median preference value and its 95% credibility intervals are obtained with function

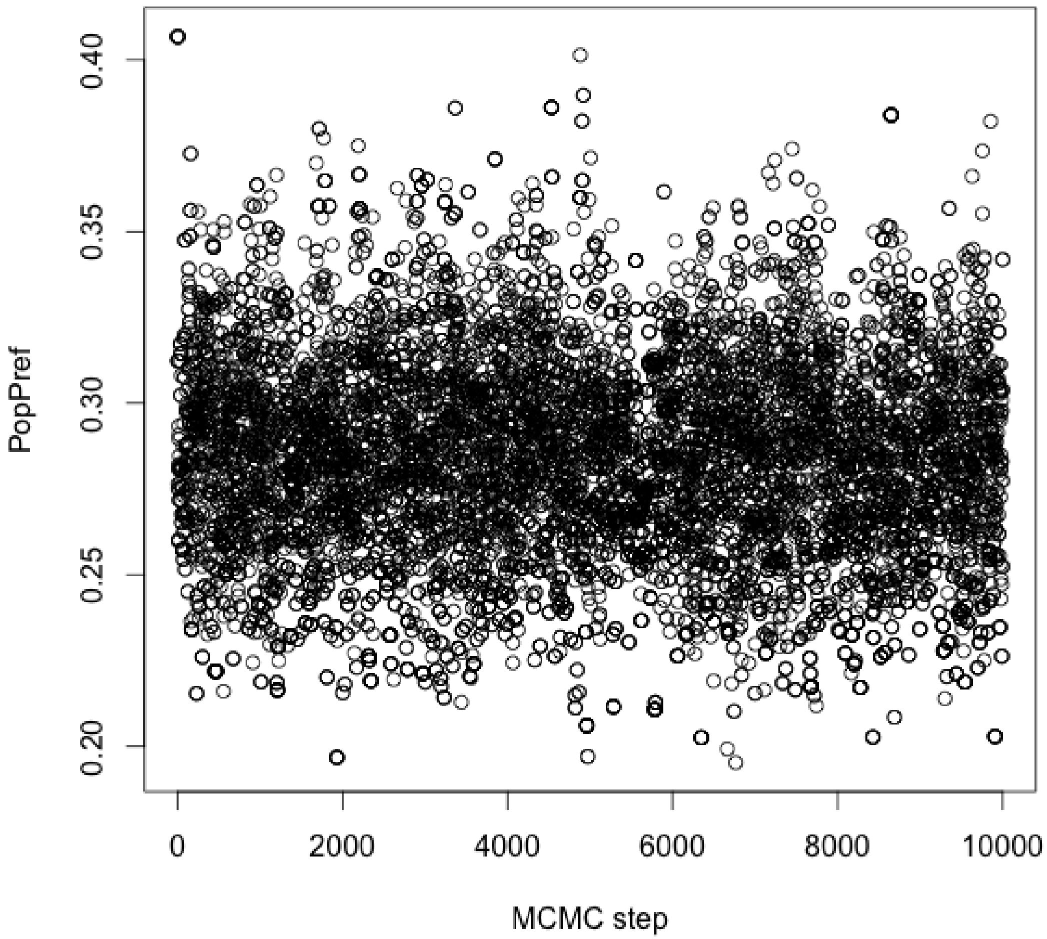

Figure 1: Supplementary R Script Figure 4. Plot of chain mixing for Bin 5

‘credibleIntervals’. Block 1 shows lower 95% CI, block 2 the median preference, and block 3 the upper 95% CI. Credible intervals for each data bin can be called using repeated runs of the following script, where ‘fgpref[[x]]’ is used to identify bins from 1 - 7 (in this case Bin 5).

```
credibleIntervals(prefres = fgpref[[5]], burn = 1000, interval = 0.95)
```

Individual preference data edited out for brevity.

```
$PopPref
          [,1]      [,2]      [,3]
[1,] 0.2679243 0.2985002 0.3312712
[2,] 0.6687288 0.7014998 0.7320757

$PopVar
    2.5%      50%    97.5%
13.50228 24.43097 43.74280
```

An identical workflow was created and run for the intensity of urbanistion (Combined Index) analysis.

SUPPLEMENTARY R SCRIPT #4

Plotting Frequency Greenspace preference data from 'bayespref' analysis

This is an R Mardkown file which contains a 'ggplot2' script for plotting urban tolerant bird preference for Frequency Greenspace bins from a 'bayespref' analysis that I ran in:

Conole, L. E. (2013). Habitat-of-origin predicts degree of adaptation in urban tolerant birds. PeerJ PrePrints. doi:10.7287/peerj.preprints.156v2. https://peerj.com/preprints/156v2

```
library(ggplot2)
```

```
fgprefs <- read.table("FG.birds.CI.txt", header = T)
fgprefs
```

```
##    bin     urban median.pref   low    up
## 1  2.0   Adapter       0.206 0.126 0.292
## 2  2.2 Exploiter       0.794 0.708 0.874
## 3  3.0   Adapter       0.279 0.222 0.347
## 4  3.2 Exploiter       0.721 0.653 0.778
## 5  4.0   Adapter       0.289 0.268 0.330
## 6  4.2 Exploiter       0.701 0.670 0.732
## 7  5.0   Adapter       0.396 0.367 0.423
## 8  5.2 Exploiter       0.604 0.577 0.633
## 9  6.0   Adapter       0.460 0.424 0.494
## 10 6.2 Exploiter       0.540 0.506 0.576
```

In the data frame 'fgprefs', column headings and the data to which they refer are as follows:

- bin refers to 7 binned intervals of Frequency Greenspace within the larger data set,
- urban refers to the two urban tolerant bird assemblages of Adapter and Exploiter,
- median.preference is the median population preference for that Frequency Greenspace bin,
- low indicates the lower 95% confidence interval around the median,
- up indicates the upper 95% confidence interbal around the median.

Using 'ggplot2' to plot the habitat preferences at landscape scale for urban adapter and exploiter birds is achieved with the following script:

```
p = ggplot(fgprefs, aes(x = bin, y = median.pref, shape = urban))
p = p + geom_pointrange(aes(ymin = low, ymax = up), size = 1.5, xlim = c(1:6))
p = p + labs(x = "Frequency Greenspace", y = "Median preference")
print(p)
```

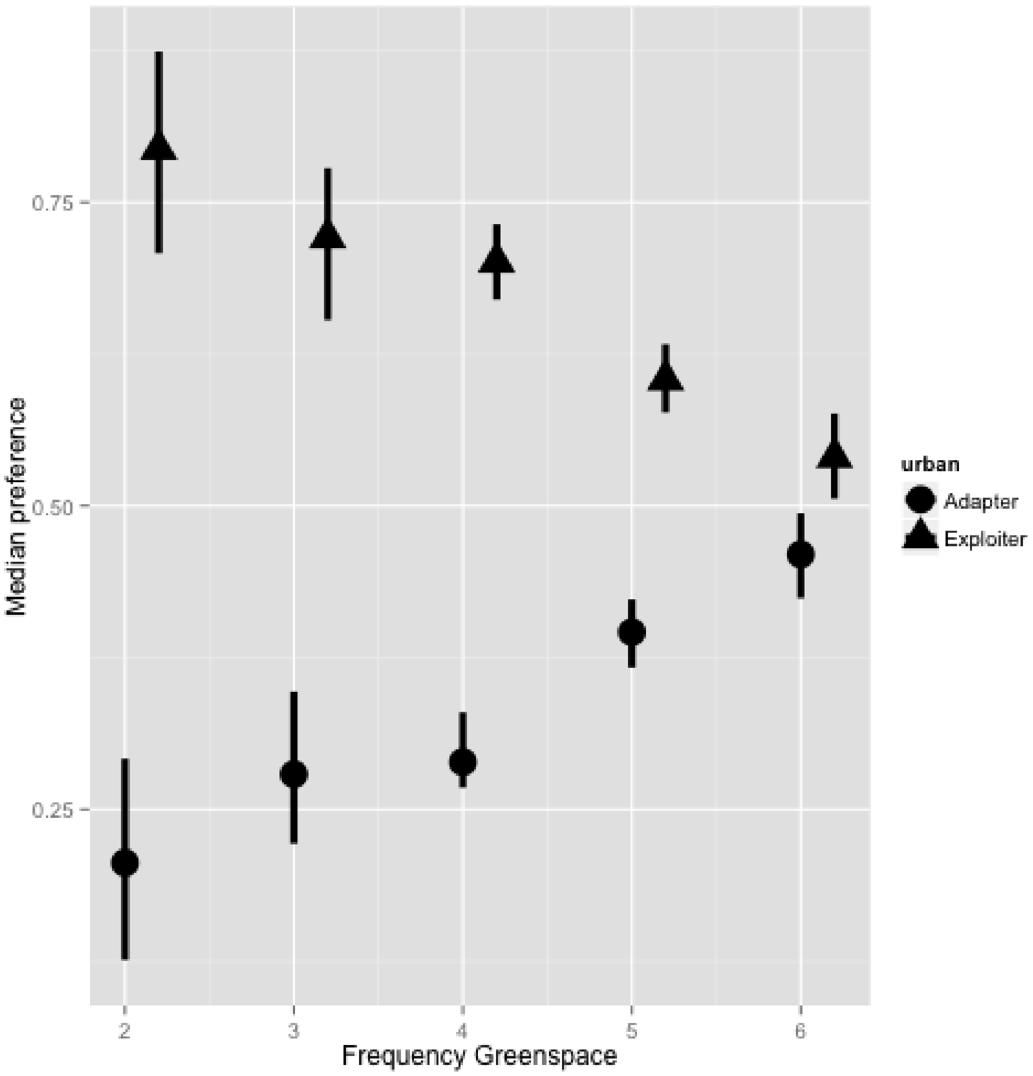

Figure 1: Supplementary R Script Figure 5. Median preference of urban tolerant birds in greenspace bins

SUPPLEMENTARY R SCRIPT #5

Script for a hierarchical agglomerative cluster analysis of 'habitat-of-origin' for urban tolerant birds

This is an R Markdown file which contains a script for a hierarchical agglomerative cluster analysis of 'habitat-of-origin' for urban tolerant birds analysis that I ran in:

Conole, L. E. (2013). Habitat-of-origin predicts degree of adaptation in urban tolerant birds. *PeerJ PrePrints*. doi:10.7287/peerj.preprints.156v2. link

Read in data from tab-delimited text file and eyeball the first few lines of the resultant dataframe.

```
eac <- read.table("EA.cluster.txt", header = T)
head(eac)
```

```
##                        Species Feral Forest Woodland Heath Scrub Urban Farm
## 1      Whitebrowed.Scrubwren     0      1        1     1     1     0    0
## 2             Brown.Thornbill     0      1        1     1     1     0    0
## 3 Yellowtailed.BlackCockatoo     0      1        1     1     0     0    0
## 4           Ganggang.Cockatoo     0      1        1     0     0     0    0
## 5     Sulphurcrested.Cockatoo     0      1        1     0     0     0    0
## 6     Blackfaced.Cuckooshrike     0      1        1     0     0     0    0
##   Grassland Air Nest.U Nest.T Nest.G Nest.C
## 1         0   0      1      0      0      0
## 2         0   0      1      0      0      0
## 3         0   0      0      1      0      0
## 4         0   0      0      1      0      0
## 5         0   0      0      1      0      0
## 6         0   0      0      1      0      0
```

In the dataframe 'eac', column headings and the data to which they refer are as follows:

- first column = species
- columns 2 - 10 = habitats-of-origin

Create a distance matrix from the dataframe, using Manhattan distance measure.

```
eac.d <- dist(eac, method = "manhattan")
```

```
## Warning: NAs introduced by coercion
```

With this script, run the cluster analysis (using Ward's method), and plot the outcome:

```
eac.w <- hclust(eac.d, method = "ward")
plot(eac.w, hang = -1, labels = eac$Species,
    + main = "Cluster dendrogram - adapters & exploiters")
```

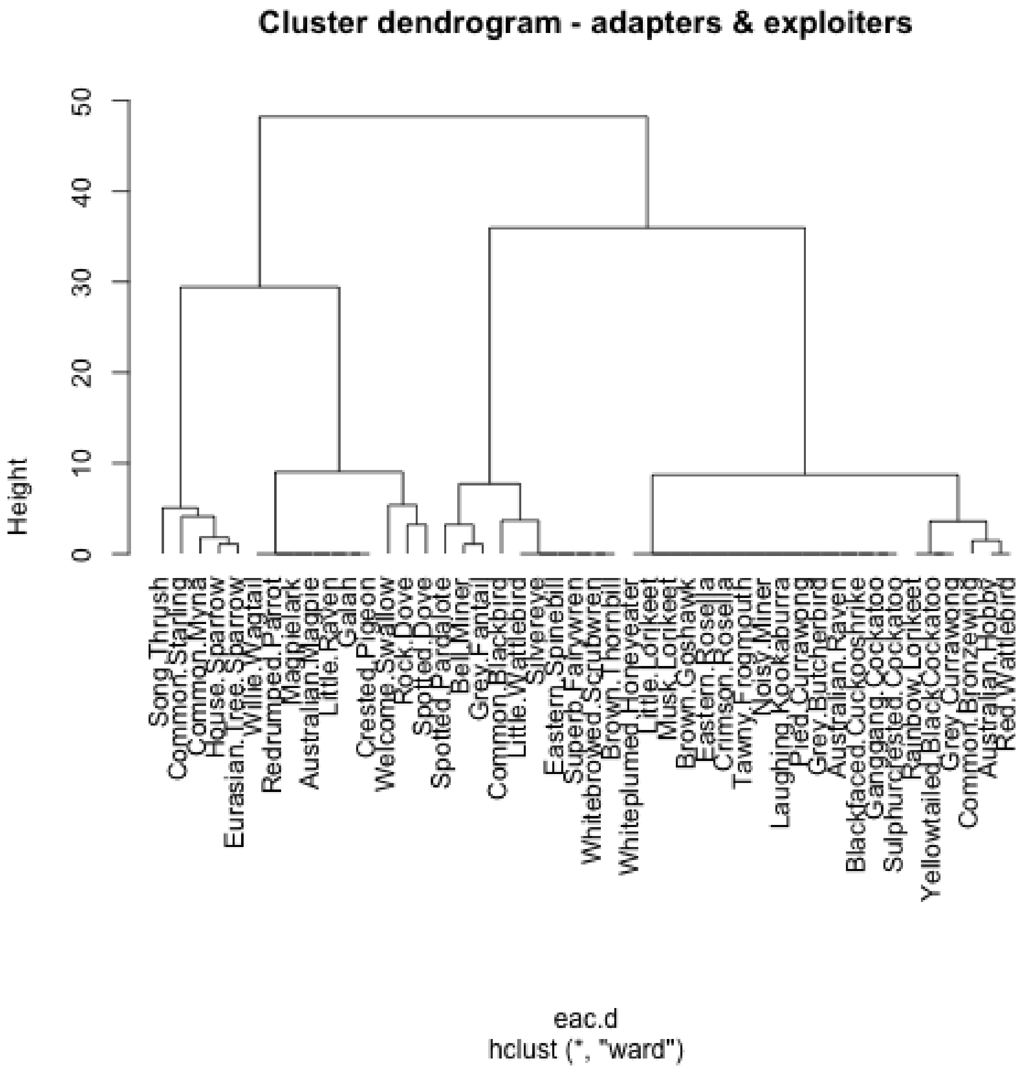

Figure 1: Supplementary R Script Figure 6. Cluster analysis - habitat-of-origin of urban tolerant birds
